# Cone-rod dystrophy can be a manifestation of Danon disease

**DOI:** 10.1007/s00417-011-1857-8

**Published:** 2012-01-31

**Authors:** Alberta A. H. J. Thiadens, Niki W. R. Slingerland, Ralph J. Florijn, Gerhard H. Visser, Frans C. Riemslag, Caroline C. W. Klaver

**Affiliations:** 1 Department of Ophthalmology, Erasmus Medical Center, P.O. Box 2040, NL-3000 CA, Rotterdam, Netherlands; 2 Departments of Clinical and Molecular Ophthalmogenetics, Netherlands Institute for Neuroscience, an institute of the Royal Netherlands Academy of Arts and Sciences, Amsterdam, Netherlands; 3 Department of Clinical Neurophysiology, Erasmus Medical Center, Rotterdam, Netherlands; 4 Bartiméus Institute for the Visually Impaired, Zeist, Netherlands; 5 The Rotterdam Eye Hospital, Rotterdam, Netherlands; 6 Department of Epidemiology, Erasmus Medical Center, Rotterdam, Netherlands

**Keywords:** Cone-rod dystrophy, LAMP2 gene, Genotype–phenotype correlations, RPE pathology, Danon disease

## Abstract

**Background:**

Danon disease is a neuromuscular disorder with variable expression in the eye. We describe a family with Danon disease and cone-rod dystrophy (CRD).

**Methods:**

Affected males of one family with Danon were invited for an extensive ophthalmologic examination, including color vision testing, fundus photography, Goldmann perimetry, full-field electroretinogram (ERG), and SD-OCT. Previous ophthalmologic data were retrieved from medical charts. The LAMP2 and RPGR gene were analyzed by direct sequencing.

**Results:**

Two siblings had no ocular phenotype. The third sibling and a cousin developed CRD leading to legal blindness. Visual acuity deteriorated progressively over time, color vision was severely disturbed, and ERG showed reduced photopic and scotopic responses. SD-OCT revealed thinning of the photoreceptor and RPE layer. Visual fields demonstrated central scotoma. The causal mutation was p.Gly384Arg in LAMP2; no mutations were found in RPGR.

**Conclusions:**

This is the first description of CRD in Danon disease. The retinal phenotype was a late onset but severe dystrophy characterized by loss of photoreceptors and RPE cells. With this report, we highlight the importance of a comprehensive ophthalmologic examination in the clinical work-up of Danon disease.

## Introduction

Danon disease is a rare genetic condition caused by mutations in the X-linked (XL) lysosome-associated membrane protein gene (LAMP2). Danon consists of triad muscle weakness, cardiomyopathy, and mental impairment. The mortality rate in males is high; the most frequent cause of death is a heart arrhythmia. Female carriers can show a mild phenotype, often restricted to cardiomyopathy [1, 2].

To date, ophthalmologic involvement has been described in only a few cases. Retinal abnormalities were reported; however, detailed work-up including psychophysical testing was lacking [1, 3–5]. It was therefore unclear which retinal cell types were affected, whether it included rods and cones, and whether the disease progressed to legal blindness. Here, we present the results of a comprehensive ophthalmologic examination in a small Danon family with cone-rod dystrophy (CRD) due to an uncommon missense mutation in LAMP2.

## Methods

### Clinical examination

A proband with Danon disease presented at our clinic with visual complaints. After his visit, we invited his three cousins with Danon for an eye examination. They all underwent an extensive examination, including best-corrected Snellen visual acuity (BCVA), refractive error, color vision testing (American Optical Hardy–Rand–Rittler test), full-field electroretinogram (ERG), fundus photography centered on the macula (TRC 50IX; Topcon, Tokyo, Japan), and spectral-domain optical coherence tomography (SD-OCT). ERGs incorporated the recommendations of the ISCEV [6]. Direct sequencing of the entire coding regions and flanking sequences of the genes LAMP2 and RPGR was performed at the Netherlands Institute of Neurosciences. The additional RPGR screening was performed to exclude other genetic causes. To the best of our knowledge, this gene is the only known causal gene for XL-CRD to date [7]. The study was approved by the Medical Ethics Committee of Erasmus Medical Center, and adhered to the tenets of the Declaration of Helsinki. The participants provided signed, informed consent for participation in the study, retrieval of medical records, and use of blood and DNA for research.

## Results

**Fig. 1:**
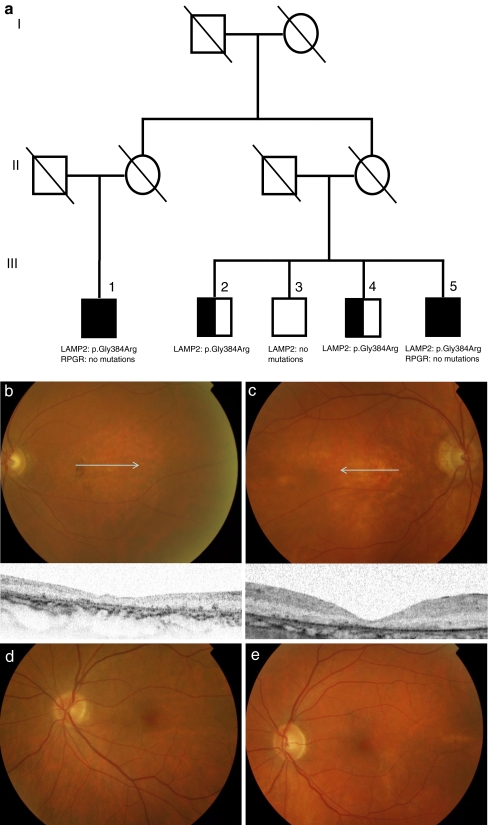

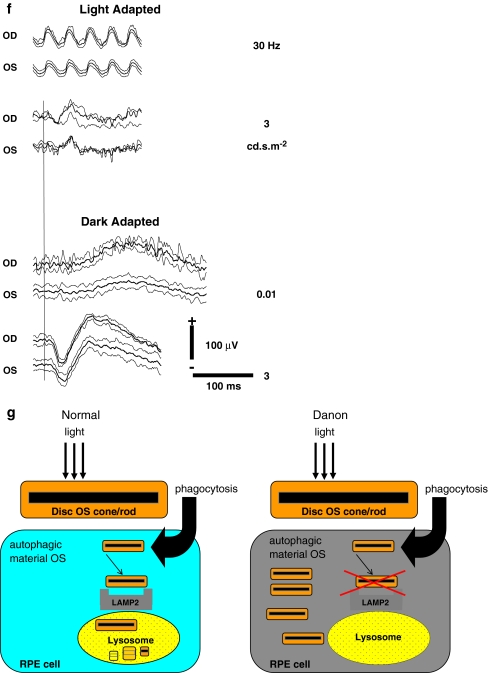
The pedigree, fundus photographs, spectral-domain optical coherence tomography (SD-OCT), electroretinogram (ERG) and a schematic representation of the localization of the LAMP2 protein in the retinal pigment epithelium (RPE) of the X-linked family with Danon disease and cone-rod dystrophy (CRD). **a** The pedigree shows two affected siblings with CRD and the mutation p.Gly384Arg in the LAMP2 gene; no mutations in the RPGR gene. *Open square*: unaffected male; *black square*: affected males with Danon disease and CRD; *half-open square*: affected males with Danon disease but without CRD; *dashed symbols* denote deceased individuals. **b** Fundus photograph of the left eye of the proband III-1, performed at age 69, showing RPE clumping and atrophy in the macula. The *arrow* denotes the position of the SD-OCT image, showing thinning of the outer segments and RPE, pigment clumping at the RPE layer and in the photoreceptor cell layer, and window defects due to atrophy of the RPE. **c** Fundus photograph of the right eye of the affected cousin III-5, performed at age 64, showing RPE atrophy in the macula. The *arrow* denotes the position of the SD-OCT image, showing a thinner but intact photoreceptor layer and thinning of the RPE cell layer. The SD-OCT cross section is not fully perpendicular. **d** Fundus photograph of the left eye of the unaffected cousin III-2, performed at age 68, showing a normal macular appearance. **e** Fundus photograph of the left eye of the unaffected cousin III-4, performed at age 66, showing a normal macular appearance. **f** Electroretinogram of proband III-1 (69 years) performed with the standard International Society for Clinical Electrophysiology of Vision (ISCEV) protocol. Replications of the responses are shown as *thin traces*, the average as *solid*. ERGs of 17 age-related normal subjects (63 ± 5 years) were analyzed in terms of amplitudes and peak latencies of the relevant components, of which the two SD criteria are mentioned below. In the dark-adapted state, the b-onset amplitudes were reduced to 80 μV and 39 μV for right and left eye respectively (normal: ≥105 μV), and the b-latencies were increased to 125 ms and 128 ms for right and left eye respectively (normal: ≤115 ms) (0.001 cd.s/m^2^). The b-a amplitudes for the 3 cd.s/m^2^ were 190 μV and 162 μV for the right and left eye respectively (normal: ≥172 μV). The a-latencies were 25 ms and 29 ms (normal ≤19 ms), and the b-latencies were 72 ms and 67 ms for the right and left eye respectively (normal: ≤62 ms). Note the reduced rod-specific response is more severely reduced in the left eye. In the light-adapted state, the b-a amplitudes were reduced to 64 μV and 43 μV for right and left eye respectively (normal ≥68 μV), and the b-latencies were increased to 41 ms and 40 ms for right and left eye respectively (normal: ≤34 ms). The diminished cone-specific function was also proven by the mildly reduced amplitudes of the cone-specific response to 30 Hz flicker stimulation (45 μV and 35 μV, for right and left eye respectively (normal ≥37 μV), and increased peak latencies to 37 ms and 38 ms for right and left eye respectively (normal: ≤33 ms). **g** Schematic drawing showing the presumed localization of the LAMP2 protein in the RPE lysosome, and the accumulation of outer segment remnants in the RPE cell in Danon disease. Abbreviation: *OS*: outer segments of photoreceptor cells.

The proband (III-1) and three of the four cousins (III-2, III-4, III-5) had Danon disease caused by a missense mutation, c.1150 G > C, leading to an amino acid change (p.Gly384Arg) in splice variant B (exon 9B) of the LAMP2 gene (reference sequence NM_013995.1; nomenclature according to http://www.hgvs.org/Mutnomen/). One cousin was unaffected (III-3), and had no LAMP2 mutation. No RPGR mutations were found in the cousins with CRD (Fig. 1a). Table 1 shows the clinical findings of this family.

**Table 1:** Clinical characteristics of the four relatives with Danon disease and a mutation in the LAMP2 gene

	LAMP2 mutation	Danon triad			CRD^#^	Ophthalmologic examination					
Family member		Mental Retardation	Muscle weakness	Cardio-myopathy		BCVA*	Color vision	Macula	Periphery	Goldmann Perimetry	ERG†
III-1	+	−	+	−	+	HM‡	All axes disturbed	Bull’s eye maculopathy	Normal	Central scotoma	Cones and rods reduced
III-2	+	−	+	−	−	1.0	Normal	Normal	Normal	Not tested	Normal
III-4	+	−	+	−	−	1.0	Normal	Normal	Normal	Not tested	Normal
III-5	+	−	+	+	+	0.05	All axes disturbed	Bull’s eye maculopathy	Pigmentary changes	Central scotoma	Cones and rods reduced

# CRD: Cone-rod dystrophy

* BCVA: best-corrected visual acuity

† ERG: full-field ISCEV electroretinogram

‡ HM: hand movements

### Case 1

Proband III-1 developed visual problems long before other symptoms were apparent. Visual decline and photophobia started at age 49 years. At age 69, BCVA deteriorated to hand movements at 1 meter. Fundus examination revealed a bull’s eye maculopathy with a normal peripheral retina, SD-OCT showed thinning of the outer segments and RPE in the macula (Fig. 1b). On ERG, cone and rod responses were both severely reduced; the latter were asymmetrically affected with more reduced rod amplitudes in the left eye and nearly normal amplitudes in the right eye. However, latency times were increased in both eyes (Fig. 1f). Muscle weakness started at age 64, and progressed rapidly to wheelchair dependency [8].

### Case 2

Cousin III-2 was 68 years old at time of last examination, and had no visual complaints. BCVA was 1.0 and ophthalmoscopy showed no retinal abnormalities (Fig. 1d). Other physical signs of Danon were weakness of the shoulder, upper arm, and distal legs.

### Case 3

Cousin III-4 was 66 years old at time of last examination, and had no ocular abnormalities (Fig. 1e). He had suffered from a generalized proximal muscle weakness in arms and legs since the age of 45, which led to wheelchair dependency. His two daughters appeared to be non-affected.

### Case 4

Cousin III-5 had had a gradually deteriorating visual acuity since his thirties to 0.05 at age 64 years. Fundus examination revealed a bull’s eye maculopathy and a peripheral salt-and-pepper retinopathy The SD-OCT showed a thinner but intact photoreceptor layer and thinning of the RPE layer (Fig. 1c). Predominantly cone responses were more severely reduced than rod responses on ERG. Goldmann perimetry revealed a central scotoma and a reduced sensitivity in the periphery. The patient suffered from muscle weakness since childhood, and became wheelchair-dependent at age 40. He developed a hypertrophic cardiomyopathy. The daughter of this patient suddenly died at the age of 29 due to cardiomyopathy.

## Discussion

In our family with Danon disease, two of the four affected males presented with all features of CRD: a progressively deteriorating visual acuity, severe color vision disturbances, a central visual field defect on perimetry, and reduced photopic and scotopic responses on ERG. The onset in this family was relatively late, i.e., middle-age, and visual acuity declined to legal blindness within 2 decades thereafter.

In the retina, strong expression of LAMP2 has been demonstrated in the lysosomes of RPE cells [5]. These lysosomes play a crucial role in the constant renewal of cone and rod outer segments. Daily, at least 2,000–4,000 packets of shedded discs are phagocytosed by the RPE, and subsequently imported and degraded into the lysosome. Mutations in the LAMP2 gene lead to RPE lysosome dysfunction, leading to accumulation of deposits and ultimately cell death. Eventually, this will lead to loss of cone and rod photoreceptor cells (Fig. 1g).

Previously described cases with ophthalmologic involvement in Danon disease had frameshift or nonsense mutations in the LAMP2 gene, leading to absence of the protein. The family of this report carried a recently identified missense mutation (p.Gly384Arg) in exon 9 of the LAMP2 gene, allowing formation of the protein [8]. An important function of exon 9 is the creation of two different splice products, LAMP2a and LAMP2b, both shown to be expressed in RPE cells [5]. Gly384Arg is located in the coding sequence for splice variant LAMP2b, jeopardizing function of only this specific isoform. The late onset of the two affected cousins in our family could be explained by the presence of this residual LAMP2 protein.

It remains intriguing why not all family members with a pathogenic mutation in the LAMP2 gene developed CRD. The non-expression in the two carriers of the p.Gly384Arg mutation (III-2, III-4) suggests that a normal function of lysosomes in the RPE can be maintained, despite a mutation in LAMP2. Other lysosomal proteins such as LAMP1 are also capable of phagocytosis of autophagic material in the RPE, and may compensate for a defective LAMP2 protein [9]. The presence of protective environmental factors in the non-affected siblings which prohibit lysosomal degradation, or a still unidentified mutation in another gene that explains CRD, are possible explanations.

In summary, this is the first description of a family with CRD and a rare missense mutation in the LAMP2 gene. CRD belongs to the clinical spectrum of Danon disease, and we therefore recommend incorporation of a comprehensive ophthalmologic examination into the regular clinical work-up of Danon patients.
